# Neem Oil (*Azadirachta indica* L.) Response Surface Methodology (RSM)-Optimized Nanoemulsions for Sensory Quality Preservation of *Oreochromis niloticus* Fillets

**DOI:** 10.3390/biology14040400

**Published:** 2025-04-10

**Authors:** Jamal Kazam, Khalid Javed Iqbal, Afshan Shafi, Usman Majeed, Maximilian Lackner

**Affiliations:** 1 Department of Zoology, The Islamia University of Bahawalpur, Bahawalpur 63100, Pakistan; jamalkazam06@gmail.com; 2 Department of Food Sciences, Cholistan University of Veterinary and Animal Sciences, Bahawalpur 63100, Pakistan; afshanshafi@cuvas.edu.pk; 3 School of Food and Biological Engineering, Jiangsu University, Zhenjiang 212013, China; majeedusman55@gmail.com; 4 Department of Industrial Engineering, University of Applied Sciences Technikum Wien, Hoechstaedtplatz 6, 1200 Vienna, Austria

**Keywords:** transmission electron microscopy, gas chromatography, minimum inhibitory concentration (MIC), time–kill, *S. aureus*, proximate analysis

## Abstract

Neem oil nanoemulsions (NO NEs) have gained attention as natural antibacterial agents due to toxicity concerns surrounding synthetic preservatives. This study aimed to prepare a response surface methodology (RSM)-optimized NO NE < 200 nm to achieve a stable dip solution to maintain the sensory quality of *Oreochromis niloticus* fillets. The NO NE achieved a stable formulation with a particle size of 160.2 ± 0.04 nm on average. The polydispersity index (PDI) was 0.1 ± 0.05, and the zeta potential was found to be 18.2 ± 0.09 mV. Gas chromatography confirmed the presence of nimbiol, nimbandiol, 6-deacetyl nimbinene, and azadirachtin in NO after ultrasonic homogenization for 10 min (alternating between 30 s rest and 30 s work time). The NE had a spherical shape with a smooth surface, as was evident from transmission electron microscopy (TEM). Furthermore, NO:PM (neem oil–potassium metabisulphite) had an MIC (minimum inhibitory concentration) value of 150 ppm, compared to 210 ppm for the NO NE alone, against *Staphylococcus aureus*. Time–kill dynamics revealed the more effective control of *S. aureus* until 72 h with NO:PM. Moreover, DNA and protein leakage also increased from 0.145 ± 0.001 to 0.769 ± 0.002 OD (optical density) and from 0.142 ± 0.002 to 0.740 ± 0.001 OD, respectively, with the co-formulation of NO:PM. Conclusively, NO:PM inhibited *S. aureus* at a lower dose compared to the NO NE alone. Time–kill dynamics revealed complete inhibition of *S. aureus* in vitro for a period of 72 h. On the other hand, a proximate analysis of *O. niloticus* fillets showed no alteration in pH, no protein loss, and juiciness/moisture retention during 30 days of storage (4 °C). Sensory panelists reported that *O. niloticus* fillets treated with NE NO had improved color, flavor, juiciness, aroma, and overall quality. These results show that NE NO is a suitable green preservative for fish and possibly other meat-based products.

## Simple Summary

Synthetic preservatives in food products have raised toxicity concerns; hence, they should be replaced with safer alternatives. Neem oil nanoemulsions (NO NEs) have shown promise as natural antibacterial agents. Here, NO NEs effectively inhibited *Staphylococcus aureus* via DNA and protein leakage due to the increased permeability of bacterial membranes caused by NEs <200 nm in size. NO NEs were made with an ultrasonicator. The main compounds found, aside from nimbiol, were nimbandiol, 6-deacetyl nimbinene, and azadirachtin. NE dip coatings on fish fillets of *Oreochromis niloticus* maintained their pH (6.65 ± 0.43), moisture (8.23 ± 0.32%), and protein content (38 ± 7%) during the storage period. Interestingly, sensory attributes such as flavor, color, and aroma were in an acceptable range. These findings suggest that NO NEs can be a safe, effective, and ecofriendly preservative for fish and other meat products.

## 1. Introduction

In global nutrition, fish plays a vital role due to its high-quality protein content with essential amino acids and omega-3 fatty acids; these are necessary to maintain health [1,2]. Nile tilapia (*O. niloticus)* is one of the most commonly farmed and consumed fish in Africa, Asia, and Latin America [3]. On a global level, its affordability, mild flavor, and ease of farming have made it an aquaculture staple food. However, like many other perishable food products, Nile tilapia fillets are highly susceptible to microbial contamination [4]. Fish is vulnerable to spoilage from bacteria like *Vibrio* spp., *Aeromonas* spp., *Escherichia coli*, *S. aureus*, *Pseudomonas* spp., and *Listeria* spp., which negatively affect flesh quality [5,6,7]. Proteolytic, lipolytic, and oxidative enzymes released from bacteria degrade the proteins, lipids, and carbohydrates of fish, and this process affects texture, flavor, color, and nutritional value [8,9], and food safety.

Synthetic preservatives (e.g., sodium benzoate, potassium sorbate, formaldehyde, and sulfites) have been traditionally used in the food industry to prevent the spoilage of perishable foods like fish fillets and ultimately extend their shelf lives [10]. However, the usage of synthetic preservatives can cause serious allergic reactions and toxicity [11,12]. Several countries have banned or restricted synthetic preservatives in foodstuffs due to their toxicity and sometimes even carcinogenicity [13,14]. Because of this awareness, plant-based preservatives like essential oils (EOs) have gained attention as alternatives to synthetic preservatives [15,16].

Plant essential oils are aromatic compounds with comparatively high volatility. They possess medicinal and antimicrobial properties [17,18]. Terpenoids, phenolics, and aldehydes in oregano, thyme, rosemary, and neem EOs have been found to exhibit broad-spectrum antimicrobial activity against *E. coli* and *S. aureus* [19,20]. Neem oil (NO, *Azadirachta indica*) is a natural preservative with antimicrobial and antifungal properties. Its addition can thereby extend the shelf life of various food products. Economically, its use supports sustainable agriculture and local industries, offering a cost-effective alternative to synthetic chemicals [21]. NO has strong antibacterial potential against the food-borne pathogen *S. aureus* [22]. However, EOs’ volatility, high required effective dose, and strong aromas have restricted their application areas [23,24]. NO is well known for its bactericidal, virucidal, fungicidal, insecticidal, and pest-repellent potential [25]. Nanocarriers, especially nanoemulsions, have larger surface areas, and they have revolutionized the application of natural bioactive substances, especially EOs. The small size of NEs produces transparent or translucent hydrophilic dispersions with extended solubility, stability, and antibacterial effectiveness [26,27]. The current study aimed to optimize NO NEs using RSM (response surface methodology) to determine the NO NEs’ in vitro antibacterial activity against *S. aureus*. Furthermore, we aimed to evaluate the potential of the NO NE to act as a natural preservative by dipping *O. niloticus* fillets and analyzing the impact of this treatment on the nutritional quality of *O. niloticus* fillets.

## 2. Materials and Methods

### 2.1. Chemicals

NO and long chain oil (canola, CA) were purchased from Karachi Essence Pvt. Ltd., Punjab, Pakistan. Ethanol and other solvents were procured from Sigma Aldrich, St. Louis, MO, USA, of analytical grade. Luria broth (LB), total plate count (TPC) agar, and sodium phosphate buffer were also purchased from Sigma Aldrich, USA. Deionized water was used for all the experiments.

### 2.2. Preparation of Nanoemulsions

First, 2% *w*/*w* Tween 80 (polysorbate, a nonionic surfactant and emulsifier) and 10% (50%:50%) oil phase NO:CA (neem oil:canola oil) in deionized water were processed via an ultrasonicator (UCD-1200, Biobase, Jinan, China). The coarse emulsion was first prepared using an Ultra-Turrax (UCD-1220, Biobase, China) at 18,000 rpm for 10 min. Subsequently, coarse emulsions were treated for 10 min via an ultrasonicator at an amplitude of 60–80% and the frequency was 0.6–0.8 kHz (UCD-1200, Biobase, China). The procedure of the NE preparation was adopted from previous work [28,29,30,31].

### 2.3. RSM for Optimized NE NEs

Response Surface Methodology (RSM) was employed to optimize the values for oil concentration, the concentration of the emulsifier, and the time of ultrasonication required to obtain narrowly distributed neem oil nanoemulsions [29]. A Box–Behnken design investigated three independent variables: the concentrations of the oil (A) and emulsifier (B), and time (C). Particle size (Y1), zeta potential (Y2), and the polydispersity index (PDI, Y3) were the dependent variables. Overall, 14 runs were used to optimize all three parameters (mean of three experimental readings). The final equation with “coded factors” is shown here:

Size= 507.813 + −199.875 × A + 9.7775 × B + −14.76 × C + −3.67 × AB + −3.6 × AC + 0.03 × BC + 55.85 × A^2^ + −0.0255 × B^2^ + 0.6795 × C^2^ (1)

PDI= 0.84625 + −0.35 × A + 0.02625 × B + −0.04525 × C + −0.012 × AB + −0.006 × AC + 0.0001 × BC + 0.105 × A^2^ + 0.00015 × B^2^ + 0.00175 × C^2^ (2)

Zeta Potential= 122.825 + −53.875 × A + −2.93 × B + −3.9125 × C + 0.4 × AB + 0.51 × AC + 0.004 × BC + 8.45 × A^2^ + 0.0915 × B^2^ + 0.0905 × C^2^ (3)

### 2.4. Transmission Electron Microscopy (TEM)

NO NE was analyzed for its morphology, shape, and dimensions using transmission electron microscopy (TEM) on a Jem 1010 (JEOL, Peabody, MA USA). The NE was diluted 10-fold and applied to a copper grid covered by a carbon film. Any excess liquid was removed by blotting it off to form a thin film, which was subsequently stained by a 1% phosphotungstic acid solution. Samples were dried for 1 min before examination under the JEOL Jem 1010 electron microscope at 64 kV accelerating voltage [32].

### 2.5. Gas Chromatography–Mass Spectrometry (GC/MS) Analysis

GC/MS (Varian 1200L, Agilent Technologies, Santa Clara, CA, USA) was performed with a DB-5 column (Agilent J&W, USA, key parameters: 30 m length, 0.25 µm film thickness, 0.25 mm column diameter). The gas chromatography was carried out at 40 °C for 2 min, followed by ramped heating from 40 to 100 °C at a rate of 8 °C per minute, and then to 230 °C at a rate of 10 °C per minute, using helium as carrier gas. Mass spectra parameters included a 70 eV ionization potential, 200 °C ion source temperature, and 30–500 Da mass range. The compounds were matched with a mass spectra library [33].

### 2.6. Determination of Minimum Inhibitory Concentration (MIC) Against S. aureus

MIC against *S. aureus* (ATCC 25923) was determined using a broth dilution assay in 96-well plates. Different concentrations of NO NE at 1 and 3% *v*/*v* (50 and 150 ppm) and PM (potassium metabisulphite) at 0.1 and 0.3% *w*/*v* (60 and 180 ppm) were added to a 96-well microplate for incubation at 37 °C. Subsequently, the samples were examined via a microplate reader at 590 nm. The lowest concentration of NO NE and NO:PM that inhibits the growth of *S. aureus* in terms of lower OD values was considered the MIC.

### 2.7. Time–Kill Dynamics

The time–kill dynamic curves were generated to evaluate the antimicrobial effectiveness of NO NE and NO:PM to counter *S. aureus*. The *S. aureus* (10 µL) was cultured on LB medium with a NO NE and NO:PM at its MIC and later incubated (37 °C) for 2 to 72 h. The bacterial colonies were counted with a marker to determine their CFU/mL. All experiments were carried out in triplicate [28].

### 2.8. Protein and Nucleic Acid Leakage

For the determination of protein and nucleic acid (NA) leakage, all samples were centrifuged (8000× *g*, 4 °C, 10 min) for pellet collection. The protein and nucleic acid release was measured via a UV–Vis spectrophotometer (UV-2600, Shimadzu, Tokyo, Japan), where the optical density (OD) at 280 and 260 nm was determined. Simultaneously, the quantity of released proteins was calculated using ultraviolet absorbance values at 280 nm against a standard curve obtained with bovine serum albumin (BSA). All experiments were performed in triplicate [34].

### 2.9. Surface Preparation

Fresh *O. niloticus* fillets were procured from the local fish market of Bahawalpur, Pakistan, kept in an ice container and transferred to the food processing lab at the Department of Food Science and Technology, Cholistan, University of Veterinary and Animal Science, Bahawalpur, Pakistan. These fillets were washed, dried, and stored for 1 day at 4 °C in sealed plastic bags. The purpose of preparing the surface was to remove any contaminants that could interfere with the adhesion of the dip.

### 2.10. Preparation of Dip-Coating

The NO NE (3% *v*/*v*) and NO:PM (3% *v*/*v*: 0.1% *w*/*v*) dipping solutions were prepared in deionized water to avoid any contamination. The *O. niloticus* fillets (65 ± 2 g) were dipped for 30 min, dried, and then refrigerated in zipper bags.

### 2.11. Application of Dipping

The fillets were dipped in 0.1% *w*/*v* PM, NO NE (3% *v*/*v*), and NO:PM (3% *v*/*v*: 0.1% *w*/*v*) for 30 min, then dried for 15 min before being refrigerated in sealed plastic bags. Undipped fillets served as control. All dipped and undipped fillets were stored separately in plastic bags in a refrigerator for 30 days (4 °C) to assess their preservation quality.

### 2.12. Proximate Analysis of the Dipped Fillets

Proximate analysis was performed at 0 and 30 days to identify the changes in proximate parameters affecting the shelf life of *O. niloticus* fillets in preserved and unpreserved mode. The analysis was carried out in triplicate to obtain the mean ± standard deviation (SD).

#### 2.12.1. Moisture

The moisture content of *O. niloticus* fillets was determined using the procedure given by [35]. A 10 g sample was transferred to a petri dish, which was then placed in a hot air oven at 100 °C until reaching a constant weight. The moisture content was determined gravimetrically:

(4) $$Moisture\;content=\frac{Loss\;of\;weight}{Weight\;of\;sample\;taken}\times 100$$

#### 2.12.2. Ash

The proximate analysis was carried out according to the standard procedures reported by [35]. The ash content of the fillets was determined in a muffle furnace (550 °C, 10 h). A 10 g fish sample was placed in a China crucible and incinerated at 550 °C until the weight stabilized. The sample was then allowed to cool in a desiccator for 15 min before its final weight was recorded. Below, the formula is shown:

(5) $$Ash\;content=\frac{Weight\;of\;Ash}{Weight\;of\;sample\;taken}\times 100$$

#### 2.12.3. pH

For the determination of pH, 1 g of fish fillet was homogenized in normal saline using an ultrasonic crusher (DLAB, D160, Shanghai, China). The pH electrodes were dipped in the crushed solution to measure the pH. The experiment was performed in triplicate [35] at room temperature.

#### 2.12.4. Protein

A sample of the *O. niloticus* fillet (10 g) was homogenized using a cell crusher (DLAB, D160, Shanghai, China), and the protein was collected by centrifugation at 10,000 rpm for 2 min (Eppendorf, Hamburg, Germany). Later, the sample was placed in a UV spectrophotometer for protein determination at 280 nm [36].

#### 2.12.5. Carbohydrates

A 0.005 g sample was added to ethanol and vortexed for maximum extraction of the carbohydrates. The extracted carbohydrate supernatant was placed into a UV-Vis spectrophotometer and measured at 270 nm. A similar method of total carbohydrate determination has already been documented [37].

### 2.13. Total Plate Count (TPC) of the Fillets

A sample of the *O. niloticus* fillet was mixed in sterile buffered peptone water solution for homogenization. The homogenate sample was inoculated on TPC (total plate count) agar to visualize any bacterial contamination [38,39]. The control and NO NE-treated fillets were washed with saline water to attain bacterial colonies. A 10 µL sample was spread on a TPC plate and incubated at 37 °C for any colony appearance. The number of colony forming units (CFU) was identified and reported as the Total Plate Count (TPC).

### 2.14. Sensory Analysis

Twenty trained sensory panelists (10 male and 10 female, average age: 23, Department of Food Science, CUVAS, Bahawalpur, Pakistan) completed the blind evaluation using a 9-point hedonic scale [40]. Each sample was offered three times to each panelist, and the answers were averaged. The samples were randomly assigned to the group. The panelists were directed to score the dipped and undipped samples based on their resemblance for the following attributes: color, flavor, juiciness, tenderness, aroma, mouth feel, and overall quality.

### 2.15. Statistical Analysis

Data were analyzed with SPSS Statistics for Windows, version 20.0 (SPSS Inc., Chicago, IL, USA) using analysis of variance (ANOVA) (*p* < 0.05). All values were presented as mean ± SD (standard deviation).

## 3. Results

### 3.1. Multiple Factor Experiments

**Figure 1:**
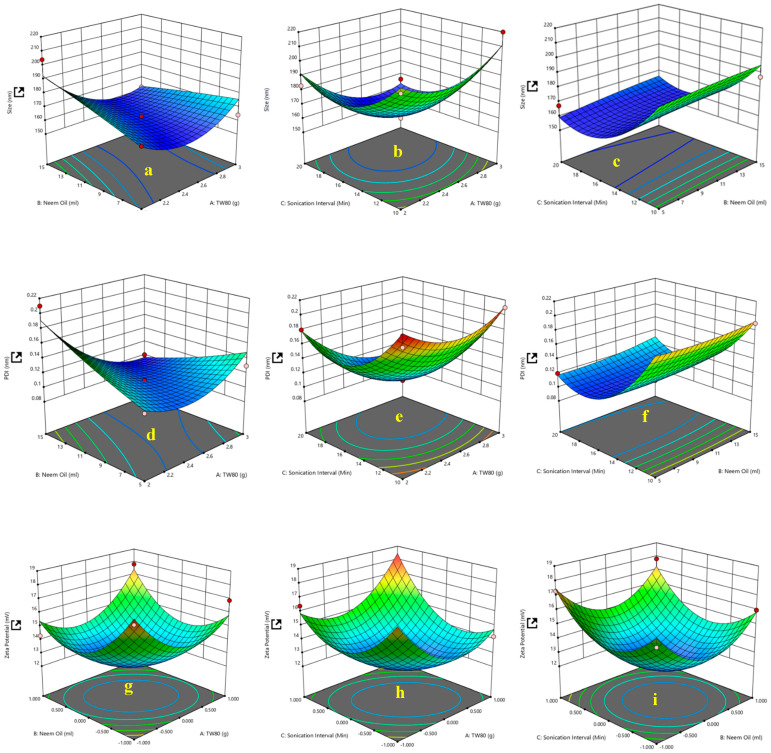
(**a**) NO (mL) versus Tween80 concentration (g); (**b**) Sonication interval (min) versus Tween80 concentration (g); (**c**) sonication interval (min) versus NO (%); (**d**) NO (mL) versus Tween80 (g); (**e**) sonication interval (min) versus NO (%); (**f**) Sonication interval (min) versus Tween80 concentration (g); (**g**) NO (%) versus Tween80 concentration (*w*/*v*); (**h**) Sonication interval (min) versus Tween80 concentration (g); (**i**) sonication interval (min) versus NO (%).

**Figure 2:**
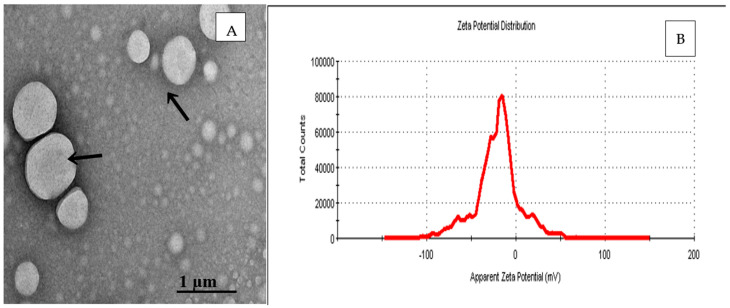
Transmission electron microscopy (TEM) image of NO NE (**A**) and Zeta Potential (**B**).

The Tween-80 concentration and NO NE dosage, as well as the sonication interval, were optimized using a Box–Behnken design of RSM, as shown in Figure 1. The droplet size of the NO NE was in the range of 160.2 ± 0.04 nm to 220 ± 0.18 nm, and the smallest droplet size of 160.2 ± 0.04 nm was observed with Tween 80 (2.5 g) and NO (10% *v*/*v* NO:CA). The PDI values were 0.1 ± 0.05 and 0.21 ± 0.05, respectively, as detailed in Table 1. The lowest PDI (0.1 ± 0.05) was achieved at 2% *w*/*w* Tween-80, 10% *v*/*v* NO, and a sonication interval of 10 min. These conditions also indicated improved NE homogeneity and stability. The zeta potential was in the range of 12.1 ± 0.01 mV to 18.2 ± 0.09 mV. The highest zeta potential value (18.2 ± 0.09 mV) was observed at Run 6 (2% *w*/*w* Tween-80, 10% NO and 10 min sonication interval), as presented in Figure 2B. The droplet size of NO NE was < 200 nm, as depicted in Figure 2A, obtained from transmission electron microscopy.

**Figure 3:**
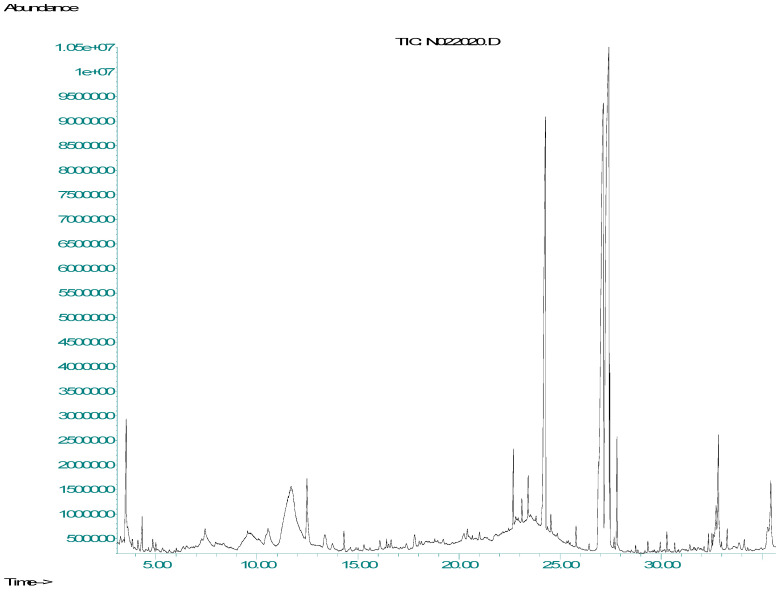
Gas chromatogram of NO NE compounds after ultrasonication. X-axis: retention time in min; Y-axis: detector signal (arbitrary units).

The gas chromatographic analysis revealed the retention time and peak areas of the different compounds in NO NE, as shown in Table 2. The retention time and peak area of nimbiol were 22.69 min and 115,540 (au, arbitrary units), while 6-deacetyl nimbin had a retention time of 24.29 min, and its peak area was 100,704 au. Similarly, 6-deacetyl nimbinene showed a retention time of 26.91 min with a peak area 114,381 au. The compound 9, 12, 15-octadecatrienoic acid (linolenic acid) had a retention time of 27.13 min with a peak area 113,306 au. The main active compound azadirachtin had the highest peak area of 116,666 au with a retention time of 27.46 min, as shown in Figure 3. Lastly, the shortest retention time was recorded as 4.33 min for nimbandiol, with the lowest peak area of 1319 au. These findings are consistent with (Alzohairy) [33], who reported azadirachtin as the most abundant active compound, which has been found to show its inhibitory effect on microbial growth through the disruption of cell walls.

### 3.2. Antibacterial Activity of NO NE and PM (Potassium Metabisulphite) Against S. aureus

The MIC values of *S. aureus* treated with NO NE and PM are illustrated in Table 3. NO NE and PM in combination showed the highest MIC value for inhibiting bacterial growth.

### 3.3. Time–Kills Dynamics

**Figure 4:**
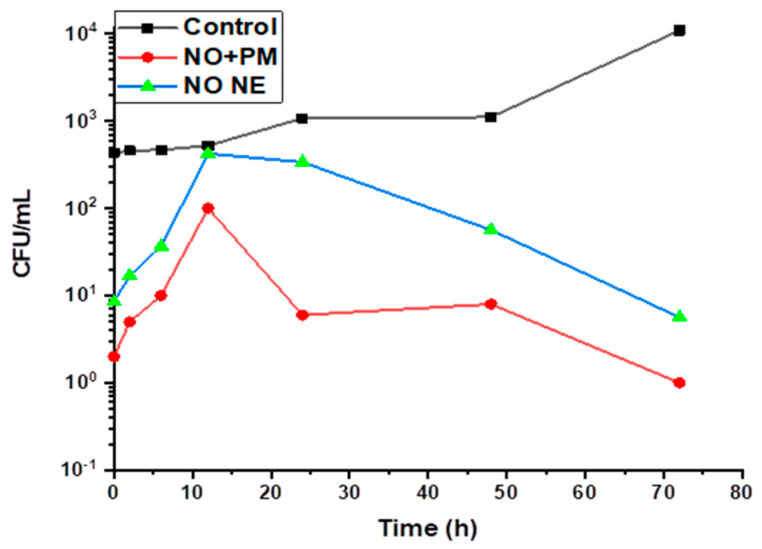
Time–kill plot for neem oil nanoemulsion (NO NE) and neem oil nanoemulsion + potassium metabisulphite (NO+PM) against *S. aureus*.

The bactericidal activity of low-dose NO NE (3% *v*/*v*) and NO + PM (3% *v*/*v*: 0.1% *w*/*v*) against *S. aureus* was examined using a time–kill dynamic experiment. Figure 4 shows the bacterial count (CFU/mL) at 0, 2, 6, 12, 24, 48, and 72 h (CFU = colony-forming units). The control group had a steady increase in growth, reaching 10^5^ CFU/mL after 72 h. In the case of NO NE, as shown in Figure 4, the bacterial count dropped to 10^1^ CFU/mL after 8 h and after 12 h increased to 10^2^ CFU/mL. After this, the bacterial count stabilized at 10^1^ CFU/mL and remained at this level until 72 h. On the other hand, NO+PM exhibited a more rapid and effective bactericidal effect. By 12 h, the bacterial count decreased to 10¹ CFU/mL, and reached 10^0^ after 72 h.

### 3.4. Protein and DNA Leakage

**Figure 5:**
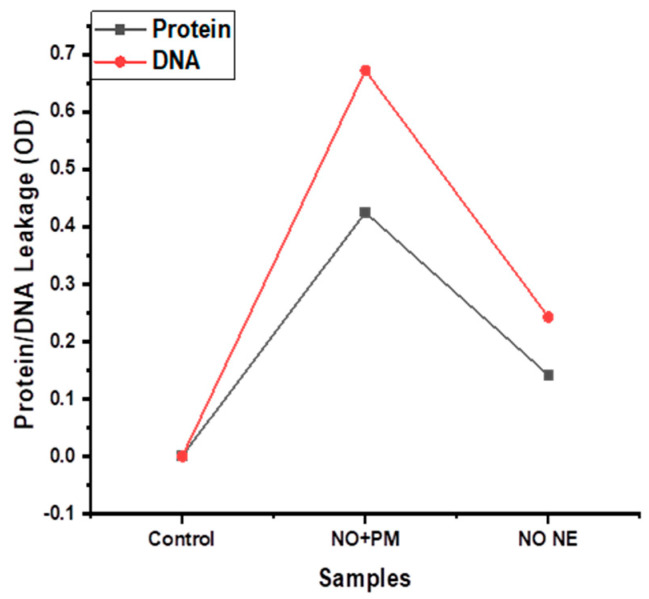
Protein and DNA leakage of *S. aureus* caused by exposure to neem oil nanoemulsion + potassium metabisulphite (NO + PM) and NO NE.

Protein and DNA leakage from *S. aureus* exposed to 3% *v*/*v* NO NE + 0.1% *w*/*v* PM were quantified using a spectrophotometer. The protein and DNA leakages were higher in the treatment group vs. the control, see Figure 5. *S. aureus* treated with NO NE exhibited protein leakage (0.13 OD), which increased to 0.36 OD when exposed to 3% *v*/*v* NO NE + 0.1% *w*/*v* PM. However, in the case of DNA, the quantity observed with 3% *v*/*v* NO NE + 0.1% *w*/*v* PM was 0.67 OD vs. the control. By contrast, the DNA leakage from NO NE-treated *S. aureus* was 0.27 OD, as shown in Figure 5.

### 3.5. Proximate Analysis

pH remained stable throughout the 30 days in all treated groups, as presented in Table 4. pH values for the 3% *v*/*v* NO NE and 3% *v*/*v* NO + 0.1% *w*/*v* PM alone consistently ranged between 5.50 and 6.65. However, the control group showed a marked decline (*p* < 0.05) in pH, reaching 5.50 ± 0.38 on Day 30. During the study period, protein levels decreased in all groups. The treatment with 0.1% *w*/*v* PM combined with 3% *v*/*v* NO NE slightly reduced the protein content from 0.40 ± 0.008 to 0.38 ± 0.06, and 3% *v*/*v* NO NE alone reduced it further to 0.37 ± 0.07. Ash content increased in the control group over 30 days, from 0.25% ± 0.34 to 1.31% ± 0.42 (*p* < 0.05), compared to treatment groups. In contrast, the combination of 3% NO NE with 0.1% *w*/*v* PM and 3% *v*/*v* NO NE alone showed a modest increase, from 1.06% ± 0.20 to 1.1% ± 0.25, respectively. All the groups maintained relatively constant moisture levels. Minor reductions in moisture were noted for 3% *v*/*v* NO NE and 3% *v*/*v* NO NE + 0.1% *w*/*v* PM, with final values between 8.20% ± 0.26 and 8.32% ± 0.42%. The decrease in moisture of the control group was slightly more pronounced, i.e., 8.12% ± 0.08 (*p* < 0.05). The minimal moisture loss in treated groups relative to the control implies a protective effect of NE treatments. By Day 30, carbohydrate levels in all treated groups had decreased slightly. However, the 3% *v*/*v* NO NE combined with 0.1% *w*/*v* PM and 3% *v*/*v* NO NE gave slightly suppressed carbohydrate levels, reaching 1.19% ± 0.07 and 1.15% ± 0.07, respectively. The 0.1% *w*/*v* PM showed a similar decline, with carbohydrate levels dropping to 1.15% ± 0.08. The control group exhibited a significant decrease in carbohydrate content from 1.20% ± 0.04 to 1.05% ± 0.08 (*p* < 0.05). Interestingly, the treatments were able to maintain or slightly lower carbohydrate concentrations, in contrast to an increase observed in the control group.

### 3.6. Total Plate Count (TPC)

**Figure 6:**
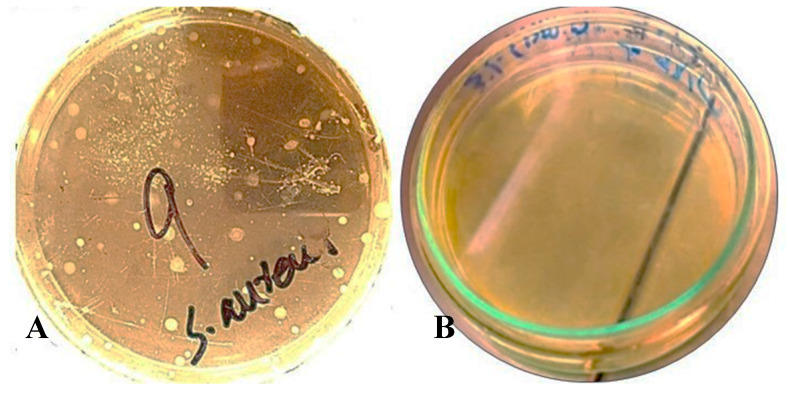
(**A**) *S. aureus* growth in the (untreated) control sample of *O. niloticus* fillet (**B**) No bacterial growth in the sample of *O. niloticus* treated with 3% *v*/*v* NO NE + 0.1% *w*/*v* PM. Diameter of the dishes: 90 mm.

The TPC of *O. niloticus* as a control and as treated with NO+PM is presented in Figure 6. The control group had clear colonies of *S. aureus* (10^4^ CFU/mL), as demonstrated in Figure 6A. However, 3% *v*/*v* NO NE in combination with 0.1% *w*/*v* PM effectively inhibited the growth of bacteria, as shown in Figure 6B. Similar studies by Safya and Rotliwala [41] demonstrated that a nano emulsion formulation improves the stability, solubility, and bioavailability of neem oil, extending its sustained antimicrobial action.

### 3.7. Sensory Evaluation

**Figure 7:**
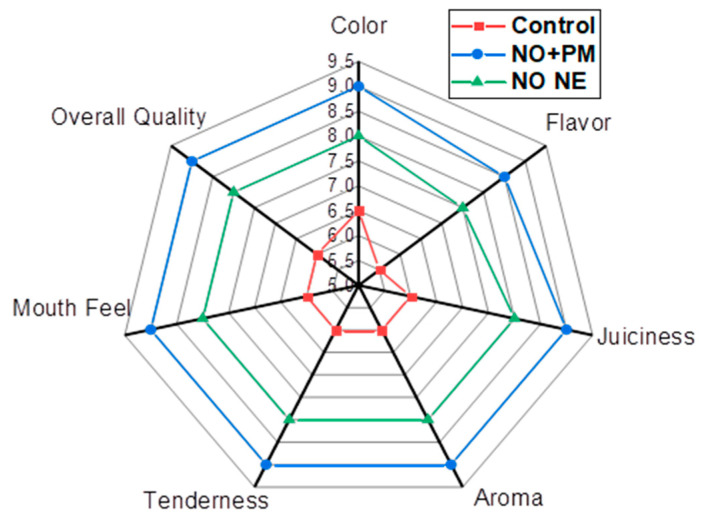
Sensory evaluation of *O. niloticus* fillets: control, NO NE, and NO:PM-treated fillets.

Sensory evaluation of *O. niloticus* after 30 days indicated significant improvements in all parameters under all treatments compared to the control (undipped sample), as displayed in Figure 7. The following observations were noted: The control sample scored 6.5 for color, while treatments with 0.1% *w*/*v* PM and 3% *v*/*v* NO NE scored 7.5 and 8.0, respectively. The highest score of 9.0 was achieved with 3% *v*/*v* NO NE + 0.1% *w*/*v* PM, which exhibited the most significantly vivid and appealing color (*p* < 0.05). Flavor scores increased from 5.5 for the control to 7.0 for 0.1% *w*/*v* PM and 7.5 for 3% *v*/*v* NO NE. The best rated (8.5) flavor was achieved with the 3% *v*/*v* NO NE + 0.1% *w*/*v* PM treatment (*p* < 0.05). juiciness–initially rated 6.0 for the control–improved to 7.0 and 8.0 with treatments of 0.1% *w*/*v* PM and 3% *v*/*v* NO NE, respectively. The treated 3% *v*/*v* NO NE + 0.1% *w*/*v* PM sample was awarded the highest score of 9.0, indicating significantly better juiciness retention (*p* < 0.05). Aroma scores for the control sample were 6.0, which increased to 7.5 when treated with 0.1% *w*/*v* PM, while 3% *v*/*v* NO NE scored 8.0. The best aroma score of 9.0 was obtained with the 3% *v*/*v* NO NE + 0.1% *w*/*v* PM treatment (*p* < 0.05). There was a gradual improvement in tenderness from the control (6.0) to 0.1% *w*/*v* PM (6.5) and 3% *v*/*v* NO NE (8.0). The sample treated with 3% *v*/*v* NO NE + 0.1% *w*/*v* PM received the significantly (*p* < 0.05) highest tenderness score of 9.0. From 2.0 in the control to 3.0, 4.0, and 5.0 with 0.1% *w*/*v* PM, 3% *v*/*v* NO NE, and 3% *v*/*v* NO NE + 0.1% *w*/*v* PM, respectively, the mouth feel score increased. This resulted in an increase in the overall quality of the samples from 6.0 for the control, 7.0 for 0.1% *w*/*v* PM, 8.0 for 3% *v*/*v* NO NE, and 9.0 for 3% *v*/*v* NO NE + 0.1% *w*/*v* PM treatment, which was significantly higher (*p* < 0.05).

## 4. Discussion

This study reveals promise for applying NO NE as a versatile formulation for improving microbial efficacy, stability, and sensory attributes. The NO NE droplet size (160.2 ± 0.04 to 220 ± 0.18 nm), PDI (0.1 ± 0.05 to 0.21 ± 0.05), and zeta potential (12.1 ± 0.01 to 18.2 ± 0.09 mV) were stable and showed excellent sustained release of the antimicrobial compounds contained therein. These results were in accordance with [29,42], who reported the 30 days’ stability of carvacrol (5-isopropyl-2-methylphenol, CAS number 499-75-2, found in oregano oil, thyme, and pepperwort) NE with a particle size of 160 nm and a PDI of 0.12. The gas chromatography confirmed the safety of the applied ultrasonication procedure, as the compounds obtained were the same as those reported in the vendor specification sheet of NO. These results were in line with [28], who reported a similar composition of clove oil (rich in eugenol) in nanoemulsions after high-pressure homogenization. Similarly to our work, [43] studied the effect of *Rosmarinus officinalis* composition on antibacterial activity and concluded that a high portion of α-pinene showed increased effectiveness against *S. aureus*.

The results on antimicrobial efficacy against *S. aureus* with 3% *v*/*v* NO NE + 0.1% *w*/*v* PM led to lower MIC values due to the synergistic action of NO and PM. A similar trend has been reported [44], where synthetic preservatives such as sodium benzoate and cumin oil have lower MIC values vs. the control. The time–kill-dynamics demonstrated that combined NO NE and PM substantially reduced kill efficacy to 10^0^ CFU/mL in comparison to the control. Similarly, sodium benzoate and cumin oil synergistically enhanced time–kill efficacy against *S. aureus* [44,45,46]. Furthermore, the protein and DNA leakages of bacteria treated with 3% *v*/*v* NO NE + 0.1% *w*/*v* PM were significantly higher than the control. These findings correlate with [34], who noted a large amount of protein and DNA leakages from bacterial cell membranes upon treatment with ultrasound in combination with thyme oil nanoemulsion. Essential oils and their active compounds interact with membrane lipids and disrupt the integrity of the phospholipid bilayer, which leads to a leakage of cytoplasmic content. The increased surface area of nanoemulsions further enhances the penetration, which allows for more effective interactions with bacterial membranes. A direct correlation between compound concentration and cellular membrane destabilization is suggested from the progressive bacterial reduction seen with increasing NO and PM concentrations. The essential oil causes structural damage to the bacterial membrane. [47].

Proximate analysis revealed that 3% *v*/*v* NO NE + 0.1% *w*/*v* PM treatment maintained pH stability as compared to the control. This formulation could prevent bacterial growth, which supports [48], who demonstrated that cinnamon essential oil nanoemulsion treatment effectively stabilizes pH in Asian seabass (*Lates Calcarifer*) fillets. The pH stability is crucial for preventing microbial growth and maintaining product quality during storage [49]. The protein content was also maintained when the fillets were treated with 3% *v*/*v* NO NE + 0.1% *w*/*v* PM, while in the control, a significant product deterioration was recorded. These findings correlate with [50], who reported that the protein content remained stable when chicken patties were treated with clove oil as a natural preservative to improve the shelf-life. Moreover, significantly decreased ash content results from 3% *v*/*v* NO NE + 0.1% *w*/*v* PM treatment indicate a retention of mineral content similar to previous work carried out by [51], who confirmed that essential oil-based nanoemulsions enhance mineral retention and utilization during the storage of *Oncorhynchus aguabonita* fillets. Further loss of moisture content was observed in the control, whereas 3% *v*/*v* NO NE + 0.1% *w*/*v* PM treatment successfully retained a higher moisture level. These findings align with work carried out by [52], where it was revealed that rosemary and olive oil nanoemulsions maintain the moisture content of fish fillets through the formation of a protective barrier. Furthermore, 3% *v*/*v* NO NE + 0.1% *w*/*v* PM treatment showed a slight decrease in carbohydrate levels, while a significant decrease in the control was noted. These findings are consistent with [53], who concluded that clove essential oil nanoemulsion could prevent carbohydrate breakdown during meat storage and inhibit harmful chemical changes. The current findings extend the understanding of NO NE’s role in maintaining nutritional attributes, which is particularly important for consumer acceptance and marketability.

The results of the TPC highlighted that the control group had visible growth of *S. aureus.* However, the 3% *v*/*v* NO NE + 0.1% *w*/*v* PM sample exhibited strong antimicrobial properties, entirely preventing the growth of bacteria. The presence of *S. aureus* in the control group correlates well with a previous study [54], in which the authors observed the lowest bacterial growth with chitosan + *Ferulago angulata* oil nanoemulsion treatment and the highest one in the control at the end of the storage period of rainbow trout fillets. The presence of a high bacterial count in the control is associated with poor hygiene and its role as a pathogenic contaminant in fish, which is hazardous to the quality and safety of fish.

Treated *O. niloticus* fillets were evaluated by a sensory panel. Significant improvements were noted for color, flavor, aroma, juiciness, and tenderness, with the highest scores observed for 3% *v*/*v* NO NE + 0.1% *w*/*v* PM and the lowest one for the control group. The highest scores of color obtained indicate that nanoemulsions can form a protective barrier that can minimize oxidative discoloration. Chitosan combined with *Hyssopus officinalis* oil nanoemulsions developed by [55] was found to effectively prevent lipid oxidation and natural color changes during the storage of shrimp (*Litopenaeus vannamei*). The result of increased flavor indicated less spoilage, and these findings are consistent with [56], who reported that grape and cinnamon essential oil nanoemulsions increase flavor while reducing the proliferation of spoilage bacteria that lead to the development of off flavors in chilled flathead mullet (*Mugil cephalus*). The enhancement of aroma suggests the inhibition of trimethylamine, similar to [55], who highlighted microbial growth and the formation of malodorous compounds like trimethylamine; these were inhibited due to the treatment of fish with Chitosan + hyssop (*Hyssopus officinalis)* oil nanoemulsion. It has been shown that nanoemulsions can reduce protein denaturation during storage by stabilizing the biochemical environment of the fish [57]. The improvement observed shows the dual advantages of NO NE of extending shelf life and improving the sensory appeal of preserved fish.

## 5. Conclusions

This study highlights the capability of NO NE as an effective antimicrobial agent and natural food preservative. The optimized formulations achieved by RSM resulted in a small average particle size, low polydispersity index, and favorable zeta potential, which demonstrated stability and efficacy against *S. aureus*, particularly when combined with 0.1% *w*/*v* PM (potassium metabisulphite). Proximate analysis revealed that NO NE in combination with PM maintained pH stability, reduced protein degradation, and minimized ash accumulation, as well as preserved moisture content during 30 days of storage. Sensory evaluation showed significant improvements in all parameters (flavor, color, juiciness, and overall quality) of *O. niloticus* fillets, with 3% *v*/*v* NO NE + 0.1% *w*/*v* PM being the most effective formulation. These results underscore the dual benefits of NO NE to prolong the shelf-life and increase product quality, providing a sustainable, natural alternative to synthetic preservatives. This approach provides promising implications for food safety and quality management in fish preservation. There is additional potential in the development of natural preservatives for foodstuff, like different meats [31], and also applications in the pharma industry, for instance. However, the large-scale production of uniformly sized NE particles could be a challenge, and their commercial application requires regulatory procedures that must be addressed.

## Figures and Tables

**Table 1:** The variation of Tween 80 concentration (TW80), NO, and sonication interval on the particle size, PDI, and zeta potential of NO NE.

	Factor 1	Factor 2	Factor 3	Response 1	Response 2	Response 3
Run	A: TW80	B: Neem Oil	C: Sonication Interval	Size	PDI	Zeta Potential
	(g)	(ml)	(min)	(nm)	(1)	(mV)
1	2	15	15	204.1 ± 0.01	0.21 ± 0.04	14.3 ± 0.07
2	3	10	20	166 ± 0.15	0.13 ± 0.01	17.5 ± 0.01
3	2.5	10	15	160.2 ± 0.04	0.11 ± 0.02	12.3 ± 0.05
4	3	10	10	220 ± 0.18	0.21 ± 0.05	14.2 ± 0.09
5	2	10	20	183.2 ± 0.09	0.18 ± 0.07	16.4 ± 0.1
6	2	10	10	201.2 ± 0.03	0.2 ± 0.04	18.2 ± 0.09
7	3	5	15	164.2 ± 0.1	0.13 ± 0.02	16.9 ± 0.11
8	2.5	5	20	167.3 ± 0.01	0.12 ± 0.03	17.3 ± 0.04
9	2.5	10	15	163.1 ± 0.03	0.11 ± 0.07	12.1 ± 0.01
10	3	15	15	162.3 ± 0.08	0.1 ± 0.05	17.8 ± 0.06
11	2	5	15	169.3 ± 0.01	0.12 ± 0.03	17.4 ± 0.05
12	2.5	15	20	163.2 ± 0.04	0.11 ± 0.01	17.8 ± 0.06
13	2.5	15	10	187.2 ± 0.11	0.19 ± 0.11	16 ± 0.02
14	2.5	5	10	194.3 ± 0.07	0.21 ± 0.05	15.9 ± 0.07

**Table 2:** Active compounds present in NO NE with their retention time and peak area.

Compound Name	CAS No.	Retention Time (min)	Peak Area (au)
Nimbiol	561-95-5	22.69	115,540
6 deacetyl nimbin	18609-16-0	24.29	100,704
6-deacetyl nimbinene		26.91	114,381
9,12,15-Octadecatrienoic acid (alpha-linolenic acid)	463-40-1	27.13	113,306
Azadirachtin	11141-17-6	27.46	116,666
Nimbandiol	78916-53-7	4.33	1319

**Table 3:** MIC of NO NE and PM against *S. aureus*.

Antibacterial Component	Minimum Inhibitory Concentration (MIC)
3% *v*/*v* NO NE and 0.1% *w*/*v* PM	210 ppm
1% *v*/*v* NO NE	50 ppm
3% *v*/*v* NO NE	150 ppm
3% PM	180 ppm
0.1% PM	60 ppm

**Table 4:** Proximate analysis of *Oreochromis niloticus* fillets after 30 days of dip coating treatments with NO NE.

Groups	pH (0 Day)	pH (30 Day)	Protein % (0 Day)	Protein % (30 Day)	Ash % (0 Day)	Ash % (30 Day)	Moisture % (0 Day)	Moisture % (30 Day)	Carbohydrate % (0 Day)	Carbohydrate % (30 Day)
3% *v*/*v* NO NE	6.80 ± 0.43	6.35 ± 0.49	40 ± 0.8	37 ± 6	0.25 ± 0.34	1.1 ± 0.25	8.35 ± 0.48	8.32 ± 0.42	1.20 ± 0.04	1.15 ± 0.07
3% *v*/*v* NO NE + 0.1% *w*/*v* PM	6.80 ± 0.43	6.65 ± 0.42	40 ± 0.8	38 ± 7	0.25 ± 0.34	1.06 ± 0.20	8.35 ± 0.48	8.23 ± 0.32	1.20 ± 0.04	1.19 ± 0.07
0.1% *w*/*v* PM	6.80 ± 0.43	6.35 ± 0.35	40 ± 0.8	35 ± 6	0.25 ± 0.34	1.13 ± 0.32	8.35 ± 0.48	8.20 ± 0.26	1.20 ± 0.04	1.15 ± 0.08
Control	6.80 ± 0.43	5.50 ± 0.38	40 ± 0.8	31 ± 6	0.25 ± 0.34	1.31 ± 0.42	8.35 ± 0.48	8.12 ± 0.08	1.20 ± 0.04	1.05 ± 0.08

## Data Availability

Data will be made available on demand.
